# Modification of Silica with Sucrose and Ammonium Fluoride Agents: A Facile Route to Prepare Supports of Iridium Catalysts for Hydrogenation Reaction

**DOI:** 10.3390/molecules29143430

**Published:** 2024-07-22

**Authors:** Ewa Janiszewska, Mariusz Pietrowski, Michał Zieliński

**Affiliations:** Faculty of Chemistry, Adam Mickiewicz University, Uniwersytetu Poznańskiego 8, 61-614 Poznań, Poland; eszym@amu.edu.pl (E.J.); mariop@amu.edu.pl (M.P.)

**Keywords:** silica, sucrose, NH_4_F modified silica, iridium catalysts, toluene hydrogenation

## Abstract

Mesoporous silica materials were synthesized using inexpensive and environmentally friendly sucrose as a porogeneous agent. It was found that the presence of sucrose and the products of its chemical transformation during synthesis (e.g., furfural polymer) significantly affected the structure of the obtained porous silica. The influence of synthesis conditions (pH, temperature, time) on the textural properties of the final materials was determined. Samples obtained in an acidic medium, at pH = 1, and treated at room temperature, yielded products with a large surface area and a narrow pore size distribution in the range of 2–5 nm, while the synthesis at pH = 8 allowed for the formation of mesoporous systems with pores in the range of 14–20 nm. To generate acidity, the silicas were modified with an ammonium fluoride solution and then used as supports for iridium catalysts in a hydrogenation reaction, with toluene as a model hydrocarbon. The influence of parameters such as specific surface area, support acidity, and iridium dispersion on catalytic activity was determined. It was shown that modification with sucrose improved the porous structure, and NH_4_F modification generated acidity. These parameters favored better reducibility and dispersion of the active phase, resulting in higher activity of the catalysts in the studied hydrogenation reaction.

## 1. Introduction

Silicon oxides, commonly named as silicas, have become useful materials in various fields due to their properties such as chemical and thermal stability, ease of surface functionalization, chemical inertness, and non-toxicity. Different synthesis methodologies, including modified Stöber, aerogel methods, self-assembly, and soft and hard templating approaches, allow for precise control over their porous properties, morphology, particle size, and uniformity [1]. Silicas prepared by these methods demonstrate significant potential for use in high-performance catalysis [2,3,4], adsorption [5,6,7], separations [8,9], food chemistry [10], drug-delivery [11], and theranostic systems [12,13,14].

Since the discovery of ordered mesoporous silicas synthesized using surfactants as templates, the templating method has been widely applied to prepare mesoporous silicas with high surface areas, tunable pore sizes, large pore volumes, and various structures and morphologies [15,16,17]. A large number of various porogeneous materials are applied in their synthesis, including ionic or neutral surfactants [18,19], triblock copolymers [20], hard templates [21], or even plant cellular structures [22]. The main drawback of these synthesis methods is the use of relatively expensive and not easily available porogeneous agents, which are usually not recovered after the synthesis is completed. Therefore, avoiding the use of such a templating agent is highly desirable and would be beneficial in terms of cost, environmental impact, and scale-up potential. Some literature reports demonstrate the use of inexpensive, harmless, and non-toxic carbohydrates as porogeneous agents in the hydrothermal synthesis of porous silica. These carbohydrates, such as glucose, sucrose, and starch, allow for the formation of mesoporous silica or the creation of mesopores in the production of hierarchical zeolites, offering an environmentally friendly alternative to conventional porogeneous agents [23,24,25,26,27,28]. The hydrothermal transformation of carbohydrates is well-documented in the literature, particularly in relation to their hydrothermal carbonization (HTC) for producing carbon spheres used in catalysis, adsorption, separation, energy storage, and drug delivery [29,30,31,32,33,34]. Several different multistep mechanisms for the formation of carbon spheres have been proposed in the literature on hydrothermal carbonization of carbohydrates [35]. Each mechanism assumes that complex saccharides initially undergo hydrolysis to monosaccharides (glucose or fructose units), which then, through dehydration, fragmentation, and a series of polymerization–polycondensation reactions, form polyfuranic [36] or aromatic-type [37] compounds, depending on the mechanism of its transformation. These reactions are facilitated by acids produced during saccharide transformation [36] or added to the aqueous solution of carbohydrates [38]. During the polymerization stage, nuclei of the newly forming polymer phase are generated and separate from the aqueous solution [36,39]. During silica synthesis, depending on the synthesis condition (temperature, time, pH), carbon polymers with different long-chain molecules are formed, onto which the inorganic precursor is condensed.

Mesoporous silicas, known for their large specific surface area, high pore volume, uniform pore size, and thermal stability, have significant potential as catalyst supports. The high surface area and pore volume enhance the dispersion of metals or metal oxides, which is crucial for many catalytic reactions. The mesopores could serve as a host for metal particles, preventing their growth and sintering at high temperatures by confining them within the channels [40,41]. Numerous studies indicate that the characteristics of supports, such as acid/base properties, morphologies, and pore structures, have a significant effect on catalytic performance. The acid/base properties of silica materials can be introduced by incorporating species such as Al, Zr, V, or Ti into their structure [42] or by functionalizing their surface with the desired functionalities such as amine, sulphonyl, thiol, or carboxyl groups [1,7,43]. We have demonstrated that modifying silica with solutions of ammonium salts at elevated temperatures, followed by subsequent calcination, generates acidic sites in the modified materials [44,45,46,47] whereas modification with solutions of alkali metal compounds results in silica with basic properties [48]. Such modification not only alters the acidity and basicity of the silica but also enhances its porosity through the formation of additional pores. The resulting materials can be used as supports for metallic catalysts or as catalysts for weak acid-catalyzed reactions.

Taking the above into account, we present a novel approach to synthesize mesoporous silica using of low-cost and environmentally friendly sucrose as an additional organic pore-forming agent. Syntheses were performed using a hydrothermal method, and the influence of reaction conditions (such as reaction temperature, pH value, and aging time) on the properties of the obtained silica samples was examined. The temperature of silica synthesis was lower than that used for synthesizing carbon microspheres. However, the increased viscosity (resulting in a jelly-like consistency) and, in some cases, changes in the color of the resulting synthesis mixture indicate the transformation of sucrose, leading to the formation of polymer chains onto which the silica precursor condensed. For comparison, syntheses in the absence of sucrose were also conducted. Selected samples with good textural properties were modified with a 0.1 M NH_4_F solution in order to introduce acidic properties to the silicas. Both NH_4_F-modified and unmodified samples were used as supports for iridium catalysts addressed to hydrogenation of toluene. The effect of catalyst texture and acidity on the activity of iridium catalysts in the hydrogenation of toluene was evaluated. To the best of our knowledge, there are no available data on such modifications of silica followed by their application as support materials for iridium catalysts in the hydrogenation processes.

## 2. Results and Discussion

**Figure 1:**
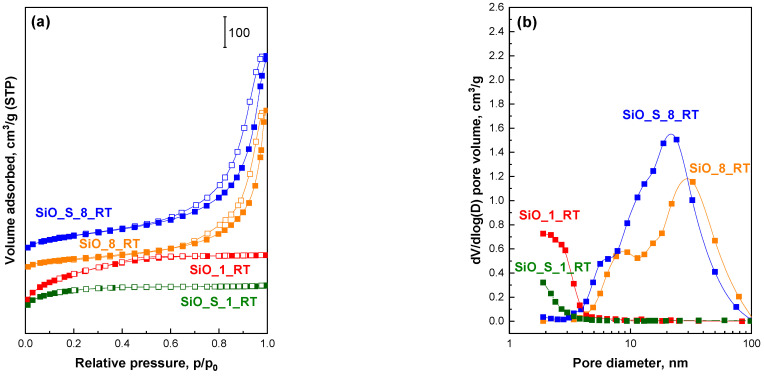
The influence of pH and the addition of sucrose on the porosity of silica obtained at 25 °C: (**a**) nitrogen adsorption–desorption isotherms, (**b**) pore size distributions.

One of the most important parameters determining the suitability of silica supports is their porous structure, including specific surface area, total pore volume, and pore size distribution. The specific surface area (SSA) was determined by the BET method (Brunauer, Emmett, and Teller), while pore volume, size, and distribution were determined by the BJH method (Barrett, Joyner, Halenda). The study was conducted on samples calcined at 550 °C for 8 h. The temperature and duration of calcination were chosen based on temperature-programmed oxidation with oxygen analysis (Figure S2). The O_2_-TPO study for the dried SiO_S_8 support revealed the presence of two peaks, at 105 and 534 °C, respectively. The first peak was due to the removal of adsorbed water, while the peak at ~500 °C was due to the oxidation of organic compounds resulting from the decomposition of sucrose transformation products. This peak was not recorded for the calcined sample. It indicated that the chosen calcination temperature of 550 °C and the period of 8 h were sufficient to remove the organic components. Table 1 and Table 2 show specific surface area values for silica supports prepared at different pH values (pH = 1, Table 1 and pH = 8, Table 2), various synthesis temperatures, and with the addition of a porogeneous agent (sucrose). Figure 1 presents nitrogen adsorption–desorption isotherms (Figure 1a) and pore size distributions (Figure 1b) obtained from the desorption branch of the isotherm for supports prepared at room temperature (RT).

Regardless of the synthesis pH, increasing the temperature of synthesis resulted in changes in specific surface areas. An elevated synthesis temperature (95 °C) for systems obtained without the addition of sucrose resulted in a decrease in the specific surface area of the supports, especially those obtained at pH = 8, where an almost 15% decrease in surface area was observed. A similar trend was observed for silicas synthesized in the presence of sucrose, with a nearly 20% decrease in the recorded SSA. Higher synthesis temperature also promoted the formation of a less homogeneous pore size distribution and an increase in pore size, see Table 1 and Table 2. Similar observations have been reported in the literature for mesoporous materials obtained in the presence of neutral surfactants [49,50]. This effect is caused by the increase in micelle radius [49] or by the weakening of hydrogen bonds between the surfactant head groups and neutral inorganic precursors with the rise in synthesis temperature [50].

Another parameter affecting the specific surface area values is the synthesis pH. During synthesis at pH = 1, supports with high specific surface area values (above 500 m^2^/g) were obtained, regardless of the synthesis temperature or the addition of a porogeneous agent. In contrast, at pH = 8, the specific surface area ranged from 150–200 m^2^/g for systems synthesized without the addition of sucrose and reached 405 m^2^/g for the support prepared with the addition of the porogeneous agent SiO_S_8_RT, see Table 2.

Based on the obtained data, the influence of sucrose on the SSA of the final systems could be determined. While for supports obtained at pH = 1 the addition of sucrose caused a decrease in surface area, for systems obtained at pH = 8, a significant (more than two-fold) increase was observed, from 182 m^2^/g for SiO_8_RT to 405 m^2^/g for SiO_S_8_RT (Table 2). A similar trend was observed for systems obtained at 95 °C. In this case, an almost twofold increase in specific surface area was also recorded, from 156 m^2^/g for SiO_8_95 to 321 m^2^/g for SiO_S_8_95. Along with changes in the specific surface area of the supports, the porous structure of the studied supports underwent significant changes. For the supports obtained at RT, the average pore size decreased from 21.1 to 9.7 nm, and the pore volume increased from 0.671 to 0.912 cm_3_/g, which was attributed to the generation of additional porosity due to the presence of an organic agent. Such substantial changes were not observed for supports obtained at pH = 1 (Table 1). In this case, the average pore size increased from 2.4 to 2.5 for systems synthesized at RT and from 3.9 to 4.4 for those synthesized at 95 °C, the influence of sucrose on porosity of silicas obtained in acidic medium was not as significant.

Samples obtained at pH = 1 exhibited type I(b) adsorption–desorption isotherms, without a clearly marked hysteresis loop. Type I(b) isotherms occur in materials with a broader pore size distribution, including wider micropores and possibly narrow mesopores (<~2.5 nm) (Figure 1b). For the SiO_1_RT support, the desorption curve did not overlap with the adsorption curve in the pressure range p/p_0_ = 0.3 − 0.6, which was related to the appearance of mesoporosity, as indicated by the presence of pores in the range of 2–4 nm, Figure 1b.

**Figure 2:**
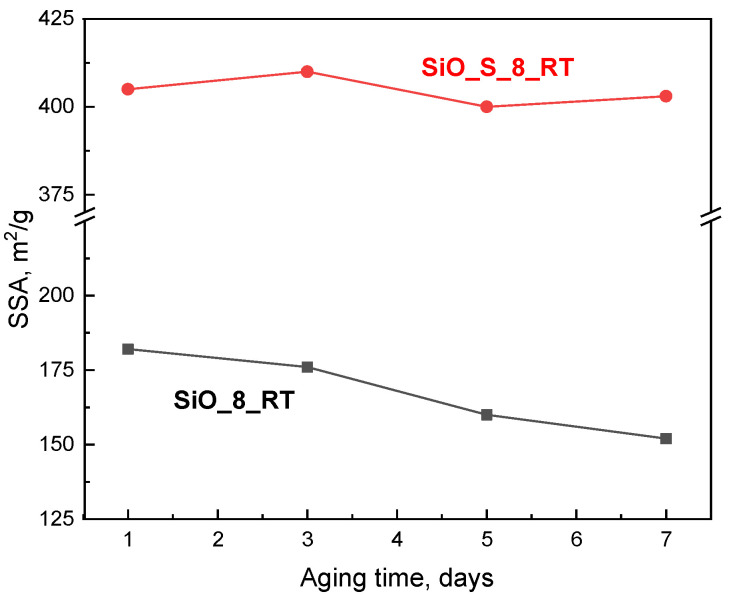
The influence of aging time on the specific surface area values for systems obtained with and without sucrose at 25 °C.

On the other hand, systems synthesized at pH = 8 exhibited type IV adsorption–desorption isotherms. This type of isotherm always indicates the presence of mesopores in the obtained samples (Figure 1a,b). Samples obtained with and without sucrose showed an isotherm with a steep increase in adsorbed volume at relative pressure p/p_0_ = 0.6–0.95, corresponding to relatively larger pore sizes and a broader pore size distribution compared to those of samples obtained at pH = 1 (Figure 2b). According to the literature, a hysteresis loop at high relative pressures (p/p_0_ > 0.8) indicates textural mesoporosity and/or macroporosity, while a sharp increase in nitrogen adsorption at p/p_0_ > 0.9 indicates significant contribution of interparticle mesoporosity [51]. For the sample SiO_8_RT, two types of pores could be observed, with maxima at ~10 and ~40 nm. Therefore, the porosity of this sample likely resulted from empty spaces formed between aggregated particles. The addition of sucrose in the initial gel is crucial for creating a homogeneous mesoporous structure, as observed for the sample SiO_S_8_RT.

In addition to synthesis temperature, pH, and the addition of sucrose as a porogeneous agent, an important parameter is the aging time of silica supports. The study was conducted for systems prepared for 1, 3, 5, and 7 days, and the results for syntheses at room temperature, pH = 8, with and without sucrose are presented in Figure 2. Additional values such as total pore volume and pore size distribution are presented in Supplementary Materials (Table S1). From the presented results, it is evident that with increasing aging time, the specific surface area for systems prepared with sucrose remained almost constant in the range of 400–410 m^2^/g, whereas for systems without sucrose, it decreased from 182 m^2^/g at 24 h to 152 m^2^/g at 7 days. This decrease could be attributed to further unfavorable transformations of the silica structure. It indicates that sucrose or the product of its transformation stabilizes the structure once formed, regardless of aging time. A similar effect was observed in the synthesis of SBA-1 material in the presence of D-fructose [52].

The textural studies allowed us to select systems for further modifications. Since increasing the synthesis temperature and extending the aging time generally led to decreases in specific surface area, and the addition of a porogeneous agent had no effect on systems obtained at pH = 1, silica systems prepared for 24 h at pH = 8 and room temperature (SiO_8_RT and SiO_S_8_RT) were chosen for further investigation. For comparison, silica prepared at pH = 1 and room temperature, characterized by the highest specific surface area of 627 m^2^/g (SiO_1_RT), was also used. To enhance readability, the symbols for the supports were shortened by removing the term “RT,” which was previously included in all support symbols.

**Modification with ammonium fluoride solution**

**Figure 3:**
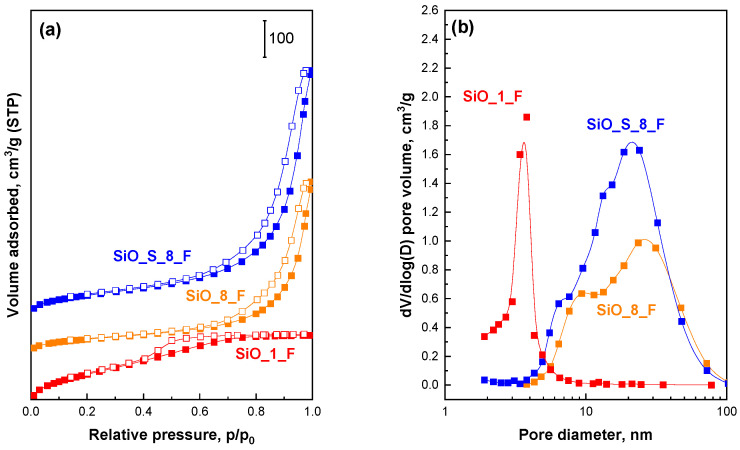
The effect of ammonium fluoride modification on the nitrogen adsorption–desorption isotherms (**a**) and the pore size distributions (**b**) of NH_4_F modified silica supports.

The selected silica supports (SiO_1, SiO_8, and SiO_S_8) were modified with an ammonium fluoride solution. The modified supports were designated by adding “-F” to the original symbol. The results for specific surface area, average pore volume, and average pore size for the pristine supports and those modified with the ammonium fluoride solution are presented in Table 2, while Figure 3 shows the nitrogen adsorption–desorption isotherms and pore size distributions for the systems modified with NH_4_F.

The modification of silica materials using an ammonium fluoride solution was performed to modify their textural and acidic properties. XPS analysis confirmed removing of fluoride ions by washing in the modified samples. Modification with a 0.1 M ammonium fluoride solution led to slight changes in the porous structure of the studied supports. The largest decrease in specific surface area (~20%) was recorded for SiO_1 (Table 3). For this material, the contribution of micropores also significantly decreased, while the external surface area (S_ext_) remained unchanged. Much smaller decreases in SSA (~5%) were recorded for the systems obtained at pH = 8, regardless of whether they were prepared with or without sucrose. For these systems, an increase in the contribution of mesopores and macropores was also observed (Table 3), with no significant changes in the shape of the hysteresis loops (Figure 3a), or the pore size distribution (Figure 3b, Table 3). The most significant changes in the nitrogen adsorption–desorption isotherm were observed for the modified SiO_1 system. In this case, a distinct hysteresis loop appeared in the p/p_0_ range of 0.4–0.8. According to the new IUPAC classification, this is characterized by a type IV(a) isotherm with an H2(b) hysteresis loop [53] (Figure 3a). The shape of the hysteresis loop (type H2(a)) can be attributed either to pore-blocking/percolation in a narrow range of pore necks or to cavitation-induced evaporation. H2(a) loops are, for instance, given by many silica gels, some porous glasses, as well as some ordered mesoporous materials (e.g., SBA-16 material). Such an isotherm is typical for mesoporous materials having a pore system for which network effects are important.

**Figure 4:**
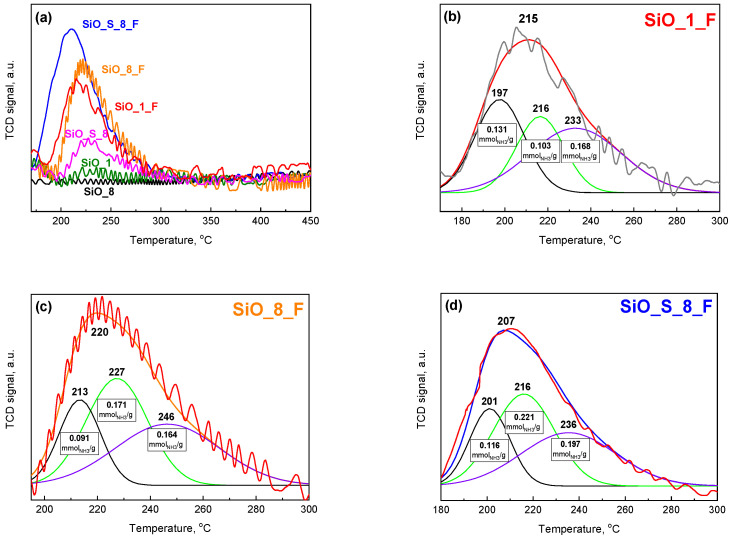
NH_3_-TPD profiles of the unmodified and NH_4_F-modified SiO_2_ samples (**a**) and the Gaussian deconvolution for NH_4_F modified supports; (**b**–**d**) signal intensity normalized to 1 g.

According to the literature data [44,46,47], modification of silica materials with a NH_4_F solution allows to generate acid centers on their surface. To determine acidity, both the starting silicas and the silicas modified with ammonium fluoride solution were analyzed by temperature-programmed desorption of ammonia (NH_3_-TPD). The results are presented in Figure 4a–d and Table 4.

Based on the NH_3_-TPD profiles, the total acidity was calculated (Table 4), and after deconvolution of the profiles (Figure 4b–d), the concentration of weak, medium, and strong acid centers was evaluated. Silica synthesized at pH = 8 (SiO_8) did not exhibit acidity, whereas the system synthesized in an acidic medium (SiO_1) showed slight acidity of 0.033 mmolNH_3_/g (Table 4), which may be due to trace amounts of chlorine not removed during washing. It was confirmed by the XPS analysis (see Supplementary Materials, Figure S3). For SiO_1 support, the signal at 198.5 eV in Cl 2p region was recorded. It was attributed to Cl from HCl. The authors of [54] have observed an analogous signal after adsorption of HCl on the Cu(100) surface. The using of sucrose in the synthesis of silica at pH = 8 (SiO_S_8) led to the generation of acid centers and the appearance of slight acidity, which may result from the formation of skeletal defects during silica synthesis due to the interaction between the silicon precursor and the sucrose. After modification of all selected silicas with an ammonium fluoride solution, the obtained materials showed a significant increase in acidity, as evidenced by the broad desorption peaks in the temperature range of 150–300 °C (Figure 4a–d).

The NH_3_-TPD profiles clearly indicated the presence of acid centers of different strengths. According to the traditional classification [44,55], the deconvoluted peaks (Figure 2b–f) could be classified into three types of acid strength: NH_3_ eluted below 215 °C (weak acid sites), within the 215–230 °C range (medium acid sites), and above 230 °C (strong acid sites). The peak present at temperatures above 230 °C was attributed to the desorption of NH_3_ from strong Brønsted and Lewis acid centers, while the low-temperature peaks were attributed to the desorption of ammonia from weak Lewis acid centers and weakly acidic silanol groups [44].

Analyzing the percentage of each type of center (Table 4), it can be concluded that in the system obtained at pH = 1 and modified with NH_4_F, weak and strong centers predominated, while in the systems obtained at pH = 8 and modified with NH_4_F, medium and strong centers predominated. This was the result of a varying contribution of different types of silanol groups formed during modification with NH_4_F, which acted as acid sites. According to Ichihashi et al. [56], the acid strength of silanol groups presented in silica material increases in the following order: terminal (isolated) groups < geminal groups < vicinal groups < silanol nests. The greater loss of surface area after modification for silica prepared at pH = 1 (Table 3) suggested the formation of a higher number of defects with OH groups located probably in close proximity, allowing for the formation of silanol nests. The modification with ammonium fluoride also changed the density of acid sites. For all systems, an increase in the density of acid sites was observed, with the largest increase (almost an order of magnitude) for the SiO_S_8_F silica.

**Figure 5:**
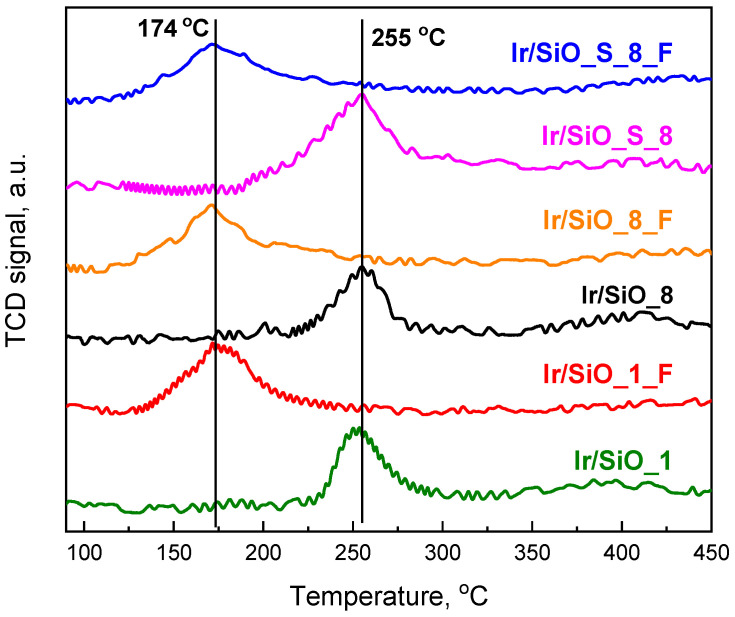
H_2_-TPR profiles of dried iridium catalyst precursors (signal intensity normalized to 100 mg).

The resulting systems, with various surface properties, were used as supports for the iridium active phase. Iridium was deposited on the supports by the impregnation method with excess solvent, using hexachloroiridic acid (H_2_IrCl_6_). The amount of active phase introduced onto the support surface was 1 wt.%. The iridium catalysts, after being dried at 105 °C for 24 h, were reduced in situ at 400 °C for 2 h (see Experimental Section). The reduction temperature was determined based on H_2_-TPR experiments (Figure 5).

For the dried catalyst precursors, two reduction regions were observed: one at 125–250 °C with a maximum around 175 °C, and another at 200–320 °C with a reduction maximum around 255 °C. A preliminary analysis of these profiles showed that the reduction maxima of the active phase precursors deposited on two types of silica—modified with NH_4_F and unmodified systems—depended on this modification. This was attributed to variations in the interaction strength between the precursors and the support surfaces, which could influence the precursor dispersion on the supports and, consequently, the distribution of the metallic phase in the final catalysts.

The H_2_-TPR profiles of iridium catalysts deposited on NH_4_F-modified silica showed a reduction maximum around ~175 °C, whereas this maximum shifted to higher temperatures (~255 °C) for catalysts on unmodified supports. Interestingly, the profiles of catalysts on supports synthesized in the presence of sucrose exhibited a similar reduction temperature to those on silica supports obtained without sucrose. The fact that the reduction of catalysts on unmodified supports occurred mainly at higher temperatures suggested a strong interaction between the Ir species and the supports, making the reduction process more difficult. Conversely, the acid sites formed in NH_4_F-modified supports appeared to interact with the iridium precursor, facilitating its reduction and affecting the dispersion and particle size of the metallic phase. For all profiles, the maximum occurred at relatively low temperatures < 300 °C, and according to data from the literature [46,57,58,59], this peak can be attributed to the reduction of highly dispersed forms of the iridium precursor to metallic iridium. This was confirmed by the calculated degree of reduction of the iridium precursor based on hydrogen consumption, which was nearly 100% for all tested systems.

As mentioned earlier, for catalysts supported on modified silicas, the main reduction peak was observed at ~175 °C. For comparison, H_2_-TPR studies of an iridium precursor impregnated on quartz sand (see Supplementary Materials Figure S4) were conducted, showing that such supported hexachloroiridic acid was reduced at 190 °C. According to the literature [60], this temperature indicates full reduction of the iridium precursor and suggests that NH_4_F-modified supports (with a reduction peak below 190 °C) provide better accessibility to the active phase, allowing for easier reduction. This issue is discussed in the section concerning the dispersion of the active phase.

Analysis of the H_2_-TPR data indicates that the temperature used for catalyst activation (H_2_, 400 °C, 2 h) was sufficient to reduce the iridium precursor to the metal phase.

**Figure 6:**
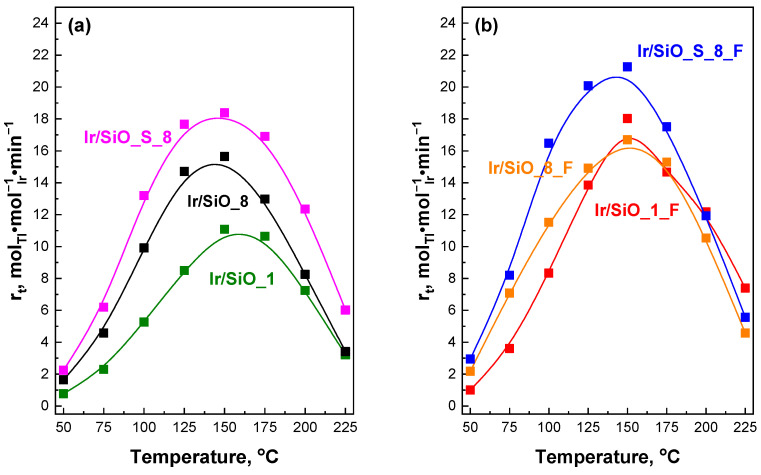
The effect of support on the apparent rate (calculated on total iridium) of hydrogenation of toluene as a function of temperature for unmodified (**a**) and modified with NH_4_F (**b**) catalysts. Activation: 400 °C, H_2_ = 100 cm^3^/min, 2 h. Reaction: c_Tl_ = 0.75 μmol/cm^3^, H_2_ = 50 cm^3^/min, activity after 20 min.

The hydrogenation reaction of toluene to methylcyclohexane was conducted in the gas phase at atmospheric pressure in a microflow reactor coupled with a gas chromatograph for catalysts reduced at 400 °C. The results for two series of iridium catalysts—on unmodified and NH_4_F-modified supports—are presented in Figure 6a,b, respectively.

The reduced iridium catalysts exhibited significant activity in toluene hydrogenation, with methylcyclohexane being the only product of this reaction. The activity increased with the reaction temperature, reaching a maximum at 150 °C. For all catalysts, a further increase in temperature caused a decrease in the activity. The decline in catalytic activity is explained in the literature by the occurrence of dehydrogenation [61] or/and cracking of methylcyclohexane formed in the studied reaction [62]. The decrease in catalytic activity may have also resulted from the loss of catalytically active sites due to surface poisoning by coke formed during the cracking of reactants. However, no by-products were observed, and as mentioned earlier, the only recorded product was methylcyclohexane. Additionally, the Wheeler–Weisz moduli (ϕ^2^η) [63] estimated for all investigated catalysts were lower than 1 within the temperature range used in this work, thus allowing to neglect diffusional limitations in our experimental conditions and their influence on the activity of the investigated catalysts. This allowed us to conclude that the only possible cause of the decrease in catalytic activity at higher temperatures was the reverse reaction, namely the dehydrogenation of methylcyclohexane.

The activity of iridium catalysts with supports not modified by ammonium fluoride (Figure 6a) depended on the type of support used and increased in the order Ir/SiO_1 < Ir/SiO_8 < Ir/SiO_S_8. A similar trend was observed for systems with silica supports modified with NH_4_F (Figure 6b), but their activity was higher compared to that of their counterparts with unmodified supports.

To elucidate the differences in activity, a correlation analysis between the activity and structure of the catalysts was conducted. This analysis focused on the activity of the catalysts in the toluene hydrogenation reaction performed at 150 °C. Firstly, the influence of the specific surface area on catalytic activity was examined. For this reason, low-temperature nitrogen adsorption–desorption measurements of iridium catalysts were performed that allowed to estimate the surface area but also average pore diameter and average pore volume. During the preparation of catalysts using the impregnation method, the active phase covered the surface of the support, thereby influencing the textural properties of the covered supports. Due to the small amount of the active phase introduced (1 wt.%), the impregnation with the iridium precursor had a negligible effect on the texture of the supports, with their surface area decreasing by 3–5% (Table 5). The impact of iridium deposition on the silica surface on the nitrogen adsorption–desorption isotherms and pore size distributions of iridium catalysts is presented in Figure S5a–d, respectively.

**Figure 7:**
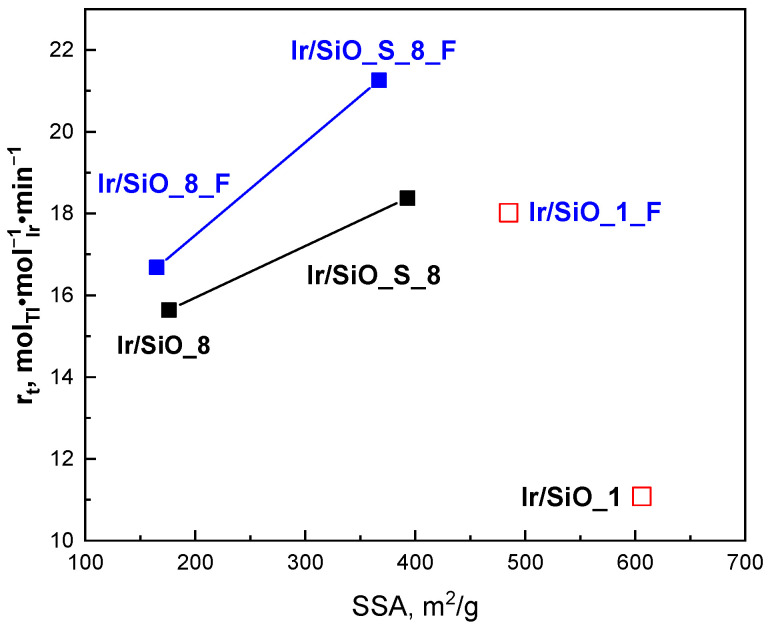
Correlations of activity in the toluene hydrogenation reaction with the specific surface area (SSA) of the catalysts for the series of catalysts on unmodified supports (black) and supports modified with NH_4_F solution (blue).

The analysis was conducted separately for the catalysts with supports obtained at pH = 8 and pH = 1, and the results are presented in Figure 7. For the systems with supports obtained at pH = 8, a linear regression line was plotted.

A correlation between activity and specific surface area was found for the series of catalysts with supports obtained at pH = 8. For both series of catalysts on modified and unmodified supports, an increase in activity was noted with an increase in specific surface area. In contrast, supports synthesized at pH = 1 resulted in catalysts with high specific surface areas, but this did not translate into higher activity in the hydrogenation process. Their activity was lower compared to that of catalysts supported on silicas synthesized at pH = 8.

The parameters that should influence catalytic activity are the size of the iridium particles and the dispersion of the active component. In our study, these were determined using the hydrogen chemisorption method, known for its high precision, especially for such low loadings of the active phase. Therefore, based on hydrogen chemisorption measurements, the average size of the iridium crystallites and iridium dispersion were determined, and the results are presented in Table 6.

The results of hydrogen chemisorption measurements indicated that the textural and acidic properties of the supports influenced the dispersion and particle size of the active iridium phase. Using silica synthesized at pH = 8 in the presence of sucrose promoted the formation of a catalyst with higher dispersion compared to that of catalysts obtained using a silica support synthesized without sucrose. This was attributed to the higher specific surface area of silica synthesized with sucrose as a porogeneous agent. Another parameter significantly affecting the size of iridium crystallites is the acidity of the support used. Supports modified with an ammonium fluoride solution exhibited much higher acidity, and catalysts prepared with their involvement (Ir/SiO_1_F, Ir/SiO_8_F, and Ir/SiO_S_8_F) showed much higher dispersions compared to those of catalysts on unmodified silicas (Table 6). The highest dispersion was observed for the catalyst Ir/SiO_S_8_F (D~76%). Although this catalyst did not possess the highest specific surface area (386 m^2^/g), it exhibited the lowest ratio of V_micro_/V_tot_ (Table 3) and the highest surface acidity of the support of 0.558 mmol_NH3_/g (Table 4). This suggests that achieving high dispersion depends not only on specific surface area but also on the presence of mesopores in the structure and the acidity of the support. Additionally, the dispersion of the active phase can be related to the reducibility of its precursor. As previously mentioned in the discussion of the H_2_-TPR results (Figure 5), supports modified with NH_4_F exhibited reduction of the active phase at lower temperatures compared to supports not treated with NH_4_F solution.

**Figure 8:**
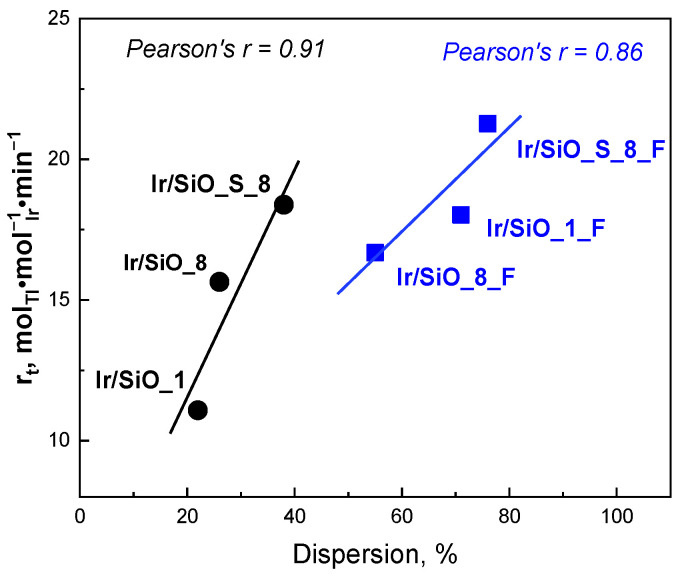
Correlations of toluene hydrogenation activity with iridium dispersion in the catalyst obtained using unmodified (black) and NH_4_F solution-modified (blue) supports.

A correlation between activity and the dispersion of the active phase was observed for both series of catalysts with unmodified and modified supports, with a better fit obtained for the former (Pearson’s r = 0.91 (Figure 8)). The graph illustrates that as the dispersion of the active phase increased, the activity in the hydrogenation of toluene increased.

**Figure 9:**
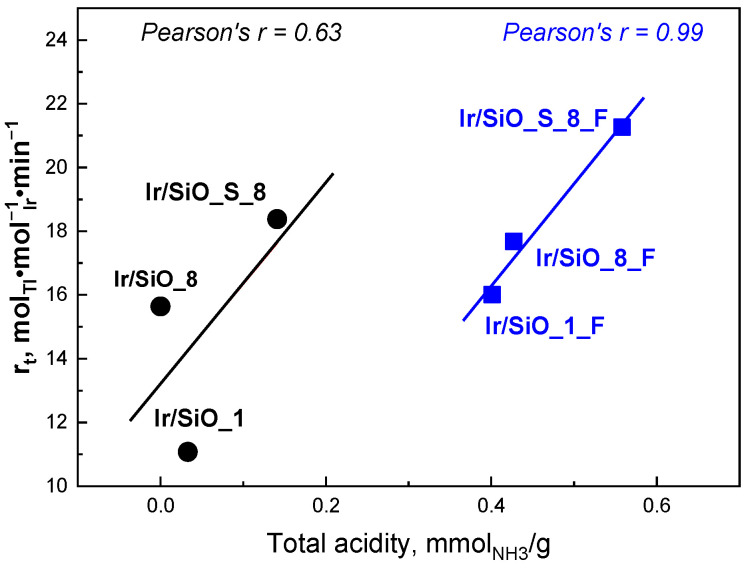
Correlations of toluene hydrogenation activity with the concentration of acid sites on the surface of the catalysts obtained with unmodified (black) and NH_4_F-modified (blue) supports.

A frequently indicated parameter influencing the activity in the hydrogenation of aromatic hydrocarbons is the concentration of acid sites responsible for the adsorption of toluene [45,64]. The correlation between catalyst activity and the acidity of their support is illustrated in Figure 9. The acidity of the supports had a significant impact on the activity of the iridium catalysts, particularly for the series obtained with NH_4_F-modified supports. The activity increased with the concentration of acid sites, showing a correlation coefficient of r = 0.99 (Figure 9). In the case of the Ir/SiO_1 catalyst, although it exhibited low acidity, its activity was much lower than that of Ir/SiO_8, which showed no acidity. This may be attributed to the fact that despite comparable dispersions of 26% and 22% for Ir/SiO_8 and Ir/SiO_1, respectively, the latter had a higher V_micro_/V_tot_ ratio of 0.113, whereas for Ir/SiO_8, this ratio was more than five times lower (Table 3).

## 3. Materials and Methods

**Scheme 1:**
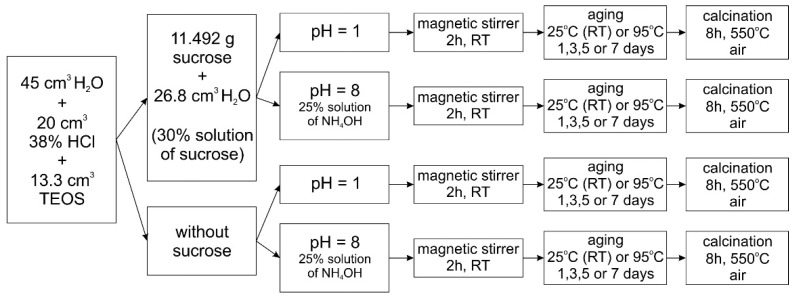
Scheme for obtaining silica supports.

Support preparation and activation:

Silica supports were obtained according to the procedure described in [28]. Tetraethoxysilane (TEOS, Sigma Aldrich- Aldrich Chemistry, Saint Louis, MO, USA) and sucrose (POCh S.A., Gliwice, Poland) were used as sources of silicon and a porogeneous agent, respectively. The syntheses of the supports were carried out at pH = 1 or pH = 8 by adding appropriate amounts of aqueous ammonia solution (25 vol.%, CHEMPUR, Piekary Ślaskie, Poland) or hydrochloric acid (38 vol.%, CHEMPUR, Piekary Ślaskie, Poland). For comparison, sucrose-free syntheses were also performed. When sucrose was used as a porogeneous agent, a 30 wt.% sucrose solution was mixed with the solution containing the silicon source and hydrochloric acid (used for hydrolysis of TEOS). The pH of the mixture was then adjusted to the appropriate value. The mixture was stirred at room temperature for 2 h using a magnetic stirrer and then the synthesis was continued under static conditions at two temperatures, 25 °C (RT) and 95 °C, for 1, 3, 5, or 7 days. The obtained products were filtered, washed with distilled water, dried, and calcined in an air flow for 8 h at 550 °C, with a temperature ramp of 3 °C/min to remove organic components. Below is a Scheme 1 of the preparation of the silica supports.

The silica supports obtained were denoted by **SiO_S_X_Y**, where **S** indicates the use of sucrose as a templating agent, **X** denotes the pH at which the silica support was obtained, and **Y** represents the ageing temperature.

Modification of the silica supports with an ammonium fluoride solution:

The obtained silica supports were activated by thermal treatment in air at 550 °C for 3 h and then were modified with a 0.1 M ammonium fluoride solution. The sample (1 g) of silica was mixed with 100 cm^3^ of an ammonium fluoride solution. The mixture was stirred under a reflux condenser at 60 °C for one hour. After treatment, the samples were filtered, washed with deionized water (3 dm^3^), dried at 105 °C for 24 h, and calcined at 550 °C for 3 h.

For example, the silica sample after NH_4_F modification was labelled as **SiO_S_X_F**, where F indicates modification with the ammonium fluoride solution. Due to the fact that all samples used for the NH_4_F modification were obtained at 25 °C, the temperature in the sample symbol was omitted.

Preparation of iridium catalysts:

Selected silica supports, both untreated and those treated with an ammonium salt solution, were used for the preparation of iridium catalysts. The iridium precursor used was a solution of hexachloroiridic acid (H_2_IrCl_6_·xH_2_O 99.98%, Aldrich) with a concentration of 0.001596 g/cm_3_ of Ir. Iridium (1 wt.%) was deposited onto silica samples using the wet impregnation method. Excess solvent was removed using a rotary evaporator, followed by drying the samples completely in a heating mantle. The impregnated supports were further dried in an oven at 105 °C for 24 h before undergoing H_2_-TPR studies.

Catalyst activation:

Before conducting measurements of low-temperature nitrogen adsorption–desorption, hydrogen chemisorption, and catalytic tests, the iridium-impregnated supports were activated in a stream of hydrogen (99.999%, Linde, Pullach im Isartal, Germany). The hydrogen flow rate was 30 cm_3_/min, and the temperature ramp was 10 °C/min. After reaching the set temperature (400 °C), the reduction of the catalyst precursor was continued for 2 h. Subsequently, the catalysts were cooled to room temperature in a hydrogen flow and then purged in an argon (99.99%, Linde, Pullach im Isartal, Germany) flow.

Support and catalyst characterization:

Textural characterization of supports calcined at 550 °C and catalysts reduced at 400 °C was obtained by low-temperature (−196 °C) nitrogen adsorption–desorption using a sorptometer ASAP 2010 manufactured by Micromeritics (Micromeritics, Norcross, GA, USA). Prior to the measurements of adsorption–desorption isotherms, the samples were outgassed at 275 °C for 6 h. The surface area was determined by the Brunauer–Emmett–Teller (BET) method, while total pore volume and average pore diameter were calculated using the Barrett–Joyner–Halenda (BJH) method from the desorption branch of the isotherm. The microporous and mesoporous surface areas were determined using the t-plot method.

Measurements of acidity using temperature-programmed desorption of the NH_3_ method (NH_3_-TPD) were conducted in a flow reactor for all silica supports on a Pulse ChemiSorb 2705, Micromeritics instrument. In a typical experiment, the sample (~500 mg) was activated in He (99.999%, Linde) for 1 h at 400 °C. The temperature ramp rate was 10 °C/min. Subsequently, the sample was cooled to 100 °C and saturated with ammonia (99.99%, Linde) for 30 min. Physically adsorbed NH_3_ was removed by purging with helium at 100 °C for 1 h. NH_3_-TPD analysis was performed in the temperature range of 100–550 °C with a ramp rate of 10 °C/min. Desorbed NH_3_ was analyzed using a thermal conductivity detector (TCD). All NH_3_-TPD profiles presented in this study were normalized to a sample mass of 1 g.

X-ray photoelectron spectroscopy (XPS) analysis of silica supports was carried out with a Kratos Axis Ultra spectrometer (Kratos Analytical, Manchester UK). The excitation source was a monochromatized aluminum X-ray source (Al Kα (1486.6 eV)) operated at 10 mA and 15 kV. The spectra were referenced to the binding energy of C (1s) (284.5 eV). Spectroscopic data were processed using CasaXPS ver. 2.3.17PR1.1software (Casa Software Ltd., Teignmouth, UK), using a peak-fitting routine with the Shirley background.

Temperature-programmed reduction with hydrogen (H_2_-TPR) measurements was carried out using a Pulse ChemiSorb 2705 instrument by Micromeritics (Micromeritics, Norcross, GA, USA). The samples were reduced in a flow of a 10% volume hydrogen mixture in argon at a gas flow rate of 30 cm_3_/min. The analysis was carried out in a temperature range from 30 °C to 500 °C, with a linear temperature ramp of 10 °C/min. A cooling mixture of isopropanol and liquid nitrogen (−75 °C) was used for water freezing. The study was performed on catalysts dried for 24 h at 105 °C. As a reference material for H_2_-TPR studies, a quartz sand (Aldrich) impregnated with hexachloroiridate(IV) hydrate (H_2_IrCl_6_·xH_2_O 99.98%, Aldrich) was used. All H_2_-TPR profiles were normalized to the same sample mass of 100 mg.

Dispersion and mean size of Ir particles were determined by the static hydrogen chemisorption method at 35 °C using a Micromeritics ASAP2010C sorptometer (Micromeritics, Norcross, GA, USA). Experimental details related to this technique are described in the Supplementary Materials (see extended experimental section) [65,66].

Catalytic test:

The toluene hydrogenation reaction was performed according to the procedure described in [46], and experimental details related to this technique are described in the Supplementary Materials (see extended experimental section). The reaction was performed at atmospheric pressure using a fixed-bed flow reactor and H_2_ (99.999%, Linde) as the carrier gas. The scheme of the setup used for toluene hydrogenation (Figure S1) and the detailed experimental procedure are presented in the Supplementary Materials and [67].

Catalytic activity was presented as the apparent rate (r_t_) calculated according to the following equation [59]:

(1) $$r_{t}=\frac{FYC}{N}$$

where *F*—total flow rate (cm^3^/min); *Y*—conversion of toluene (%); *C*—concentration of toluene in the feed (mol_Tl_·cm^3^), and *N*—iridium content (mol_Ir_) in the sample.

## 4. Conclusions

A series of silica supports were prepared under various conditions: at two pH levels (pH = 1 and pH = 8), different temperatures (25 °C or 95 °C), and varying aging times (1, 3, 5, and 7 days), with or without the addition of sucrose as a porogeneous agent. The supports with the most favorable physicochemical properties (SiO_1, SiO_8, and SiO_S_8), along with their NH_4_F-modified counterparts, were used as supports for iridium catalysts.

The results indicated that sucrose could be used effectively as a porogeneous agent for the formation of porous silica. The most significant impact of sucrose on the textural properties of the resulting porous silica was observed at pH = 8, where it led to a more than 50% increase in the specific surface area compared to that of systems prepared without sucrose. Modification of silica with an ammonium fluoride solution resulted in structural changes leading to the formation of a better porous structure. Although the specific surface area slightly decreased, there was an increase in the contribution of mesopores. The NH_4_F modification also increased the acidity of the supports, which positively influenced the activity of the obtained iridium-supported catalysts in the hydrogenation of toluene. Using fluoride-modified silica materials with a large surface area, large pore sizes and relatively high acidity as supports for Ir catalysts resulted in catalysts with higher Ir dispersion compared to that of those made with unmodified silicas. This has a crucial impact on the activity in the hydrogenation of toluene.

Our results indicate that among the parameters affecting catalytic activity (specific surface area, porosity, dispersion of the active phase, and surface acidity), the most important factors were the porous structure, dispersion of the active phase, and acidity. These parameters were most pronounced in the systems obtained on NH_4_F-modified silica supports.

## Figures and Tables

**Table 1:** Physicochemical characterization of the supports obtained at pH = 1 without and with sucrose.

Symbol of Sample	Temperature of Synthesis, °C	SSA, m^2^/g	BJH Desorption Cumulative Pore Volume, cm^3^/g	BJH Desorption Average Pore Diameter, nm
SiO_1_RT	25	627	0.199	2.6
SiO_1_95	95	555	0.695	3.9
SiO_S_1_RT	25	506	0.075	2.5
SiO_S_1_95	95	526	0.704	4.4

**Table 2:** Physicochemical characterization of the supports obtained at pH = 8 without and with sucrose.

Symbol of Sample	Temperature of Synthesis, °C	SSA, m^2^/g	BJH Desorption Cumulative Pore Volume, cm^3^/g	BJH Desorption Average Pore Diameter, nm
SiO_8_RT	25	182	0.671	21.1
SiO_8_95	95	156	0.743	24.3
SiO_S_8_RT	25	405	0.925	9.7
SiO_S_8_95	95	321	1.284	15.7

**Table 3:** Physicochemical characterization of the supports modified with ammonium fluoride.

Symbol of Sample	SSA, m^2^/g	S_ext_, m^2^/g	BJH Desorption Cumulative Pore Volume, cm^3^/g	V_tot_ Single Point Total Pore Volume at p/p_0_ = 0.99, cm^3^/g	BJH Desorption Average Pore Diameter, nm	V_micro_, cm^3^/g	V_meso_, cm^3^/g	(V_micro_/V_tot_) ·100
SiO_1	627	457	0.199	0.344	2.6	0.039	0.305	11.3
SiO_1_F	503	460	0.329	0.385	3.2	0.014	0.379	3.6
SiO_8	182	157	0.671	0.397	21.1	0.008	0.389	2.0
SiO_8_F	171	161	0.765	0.576	22.0	0.005	0.571	0.8
SiO_S_8	405	375	0.925	0.881	9.7	0.008	0.873	0.9
SiO_S_8_F	386	262	1.109	0.913	12.3	0.006	0.907	0.6

S_ext_—external surface area from the t-plot method, m^2^/g; V_tot_—single point total pore volume at p/p_0_ = 0.99, cm^3^/g; V_micro_—micropore volume from the t-plot method, cm^3^/g; V_meso_—mesopore volume, cm^3^/g.

**Table 4:** Acidic properties of silicas modified with NH_4_F and the initial unmodified silica calcined at 550 °C.

Support	Total Acidity, mmol_NH3_/g	Density of Acid Sites, mmol/m^2^(·10^−4^)	Fraction of Acid Centers of Different Strengths, %		
			Weak	Medium	Strong
SiO_1	0.033	1.8	11	74	15
SiO_1_F	0.401	7.9	33	26	42
SiO_8	0	0	0	0	0
SiO_8_F	0.427	25.0	21	40	39
SiO_S_8	0.141	3.5	20	45	35
SiO_S_8_F	0.558	14.0	21	40	35

**Table 5:** Specific surface area of the supports and iridium catalysts.

Sample	SSA, m^2^/g	
	Support	Iridium Catalyst
Ir/SiO_1	627	606
Ir/SiO_1_F	503	485
Ir/SiO_8	182	176
Ir/SiO_8_F	171	165
Ir/SiO_S_8	405	393
Ir/SiO_S_8_F	386	367

**Table 6:** Hydrogen chemisorption analysis and toluene hydrogenation activity of iridium catalysts reduced at 400 °C.

Sample	Hydrogen Chemisorption Data for Ir/Support Catalysts ^(a)^					Toluene Hydrogenation ^(c)^ at 150 °C r_t_, 1/min
	Volume Adsorbed, cm^3^/g			Dispersion, %	Average Size of Ir particle ^(b)^, nm	
	H_t_	H_irr_	H_r_	D		
Ir/SiO_1	0.48	0.13	0.35	22	5.0	11.1
Ir/SiO_1_F	0.66	0.44	0.22	71	1.8	18.0
Ir/SiO_8	0.40	0.15	0.25	26	4.3	15.6
Ir/SiO_8_F	0.57	0.32	0.25	55	2.0	16.7
Ir/SiO_S_8	0.48	0.22	0.26	38	2.9	18.4
Ir/SiO_S_8_F	0.73	0.44	0.29	76	1.5	21.3

^(a)^ Dispersion and average size of Ir particles were determined by H_2_ chemisorption—H_t_—total adsorbed hydrogen; H_r_—reversibly adsorbed hydrogen; H_irr_—irreversibly adsorbed hydrogen; D_t_—dispersion calculated from irreversibly adsorbed hydrogen. ^(b)^ The average size of iridium particles (in nm) calculated from the amount of irreversibly adsorbed hydrogen. ^(c)^ Catalytic activity expressed as apparent rate (r_t_)—see the Experimental Section and Supplementary Materials.

## Data Availability

The data presented in this study are available on request from the corresponding author.

## Supplementary Materials

The following are available online at https://www.mdpi.com/article/10.3390/molecules29143430/s1, Detailed experimental procedure—H₂ chemisorption analysis toluene hydrogenation reaction and temperature-programmed oxidation (O_2_-TPO) analysis; Figure S1: A scheme of the setup for catalytic hydrogenation of toluene; Figure S2: O_2_-TPO profiles of SiO_S_8 dried (105 °C for 24 h) and calcined (8 h at 550 °C) supports (signal intensity normalized to 100 mg); Figure S3: XPS spectrum of SiO_1 support in Cl 2p region., Figure S4: H_2_-TPR profile of iridium precursor (H_2_IrCl_6_·xH_2_O, 99.995%, Aldrich) supported on the quartz sand—1 wt.% of Ir, Figure S5: The effect of iridium deposition on the silica surface on the nitrogen adsorption–desorption isotherms (a,b) and the pore size distributions (c,d). Table S1: Physicochemical characterization of the supports obtained at pH = 8 without and with sucrose prepared for 1, 3, 5, and 7 days.
