# Supplementary material for: Modification of Silica with Sucrose and Ammonium Fluoride Agents: A Facile Route to Prepare Supports of Iridium Catalysts for Hydrogenation Reaction
Source: Molecules. 2024 Jul 22;29(14):3430. doi: 10.3390/molecules29143430 (PMC11279784; doi:10.3390/molecules29143430)
Supplement: Supplementary file 1 [file molecules-29-03430-s001.zip › molecules-3102776-supplementary.pdf]

Modification of Silica with Sucrose and Ammonium Fluoride Agents: A Facile Route to Prepare Supports of Iridium Catalysts for Hydrogenation Reaction

Ewa Janiszewska, Mariusz Pietrowski and Michał Zieliński *

Faculty of Chemistry, Adam Mickiewicz University, Uniwersytetu Poznańskiego 8, 61-614 Poznań, Poland; eszym@amu.edu.pl (E.J.); mariop@amu.edu.pl (M.P.)
* Correspondence: mardok@amu.edu.pl

Extended experimental section:

H_2 chemisorption analysis

The samples were evacuated for 10 min at room temperature, then at 360 °C for 30 min, followed by reduction in hydrogen flow (40 cm^3/min) at 360 °C for 30 min and evacuation at 360 °C for 60 min. The additional treatment in the flow of hydrogen was aimed to clean the sample from impurities that could adsorb on the sample surface during its contact with atmospheric air. Chemisorption of hydrogen was carried out at 35 °C, and the isotherms were determined using 5 different pressures in the range of 12–40 kPa, by assuming the stoichiometry of one hydrogen atom per one surface iridium atom (Ir_s).

The metallic surface area *(S)*, expressed in m^2/g_{Ir}, was calculated from the following equation [1]:

$$S = \frac{v_m \cdot N_A \cdot n \cdot a_m \cdot 100}{22414 \cdot m \cdot wt}$$

where v_m -volume of adsorbed hydrogen expressed in cm^3, N_A - Avogadro's number ($6.022 \cdot 10^{23}$/mol), *n* - chemisorption stoichiometry (*n*=2), a_m- surface area (m^2) occupied by one iridium atom, *m*- sample weight (g), *wt* - metal loading (%).
The dispersion of iridium (D) can be expressed as $D = Ir_s/Ir_t = H/Ir_t$ (where Ir_t – total number of iridium atoms) and was calculated from the formula:

$$D = \frac{S \cdot M}{a_m \cdot N_A}$$

where *S* is metallic surface area, *M* is iridium atomic weight, N_A is Avogadro's number and a_m is the surface covered by one iridium atom.
The size of iridium crystallites (P) was calculated, assuming that the metal particles are spherical, using the formula:

$$P = \frac{6000}{S \cdot q}$$

where: *q* - density of metal (iridium), g/cm^3, *S* - specific surface area of iridium, m^2/g_{Ir}.

[1] Bergeret G., Gallezot P., Handbook of Heterogeneous Catalysis, G. Ertl, H. Knözinger, J. Weitkamp, Viley Weinheim, **1997**.

Toluene hydrogenation reaction

Freshly dried catalyst (25 mg) was loaded into the reactor and reduced in situ under a flow (100 cm^3/min) of pure hydrogen at 400 °C for 2 h before starting the reaction. After reduction, the temperature was lowered to 50 °C. The reaction mixture obtained by passing hydrogen (50 cm^3/min) through a saturator filled with toluene (Aldrich) equilibrated at 10 °C was directed to the reactor. The concentration of toluene in the feed was stable and equal to 0.75 µmol/cm^3. The catalysts were heated at the rate of 10 °C/min in a flow of pure hydrogen and the catalytic activities were measured at temperatures between 75 °C and 225 °C in several steps over the same catalyst. The reaction was carried out for 20 min at each temperature and the products were analyzed every 10 min using a manually sampling system. Relative Molar Factors for toluene and methylcyclohexane were 116 and 120, respectively [2]. These coefficients were used to recalculate peak areas into molar quantities. Toluene was dosed using six-way valve that enabled the mixture to bypass the reactor. This procedure was applied in order to estimate concentration of toluene.

The reaction products were analysed on a gas chromatograph equipped with a capillary column RESTEK MXT-1. The identification of toluene and methylcyclohexane was carried out using a TCD detector.

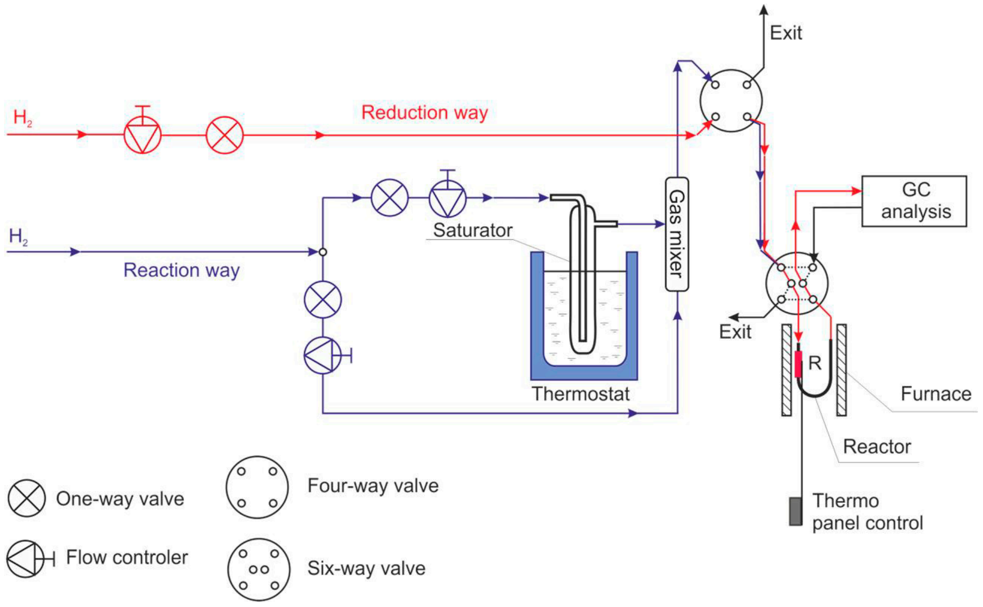

Figure S1. A scheme of the setup for catalytic hydrogenation of toluene.

[2] Dietz, W.A. Response Factors for Gas Chromatographic Analyses. *J. Chromatogr. Sci.* **1967**, 5(2), 68–71. doi:10.1093/chromsci/5.2.68.

Temperature-programmed oxidation **(O_2-TPO)**

Temperature-programmed oxidation with oxygen (O_2-TPO) measurements were carried out using the Pulse ChemiSorb 2705 instrument by Micromeritics. Samples were oxidized in a flow of a 10% volume O_2 mixture in helium at a gas flow rate of 30 cm³/min. The analysis was carried out in a temperature range from 30°C to 750°C, with a linear temperature ramp of 5°C/min. The study was performed on support dried for 24 hours at 105°C and calcined for 8h at 550°C. All O_2-TPO profiles were normalized to the same sample mass of 100 mg.

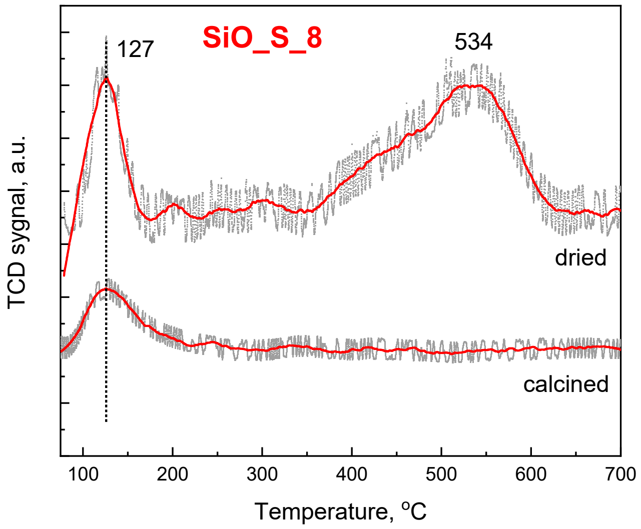

Figure S2. O_2-TPO profiles of SiO_S_8 dried (105°C for 24 hours) and calcined (8 hours at 550°C) supports (signal intensity normalized to 100 mg).

Table S1. Physicochemical characterization of the supports obtained at pH = 8 without and with sucrose prepared for 1, 3, 5, and 7 days.

Symbol of sample	Time of synthesis, days	SSA, m^2/g	BJH desorption cumulative pore volume, cm^3/g	BJH desorption average pore diameter, nm
SiO_8_RT	1	182	0.671	21.1
	3	176	0.885	21.6
	5	160	0.921	19.8
	7	152	1.008	22.4
SiO_S_8_RT	1	405	0.925	9.7
	3	410	0.900	9.3
	5	400	0.912	9.5
	7	403	0.892	8.1

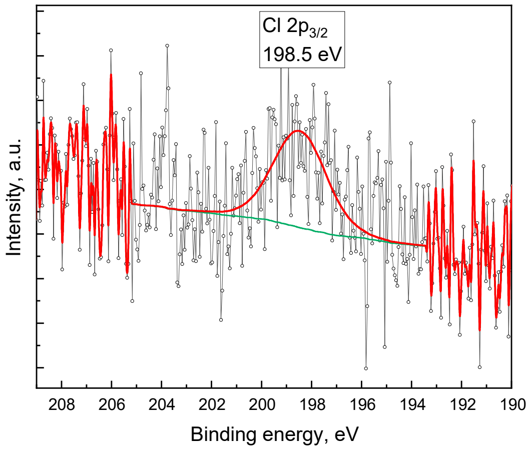

Figure S3. XPS spectrum of SiO_1 support in Cl 2p region.

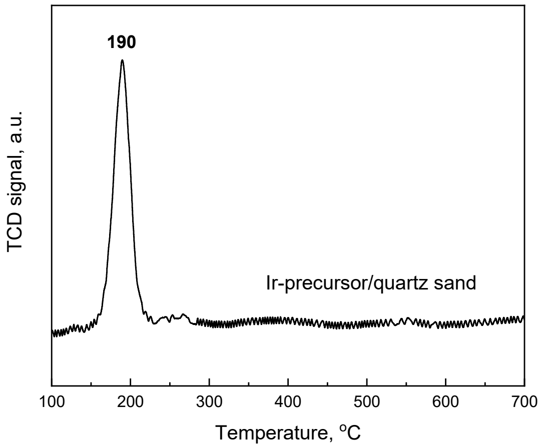

Figure S4. H_2-TPR profile of iridium precursor ($H_2IrCl_6 \cdot xH_2O$, 99.995%, Aldrich) supported on the quartz sand - 1 wt.% of Ir.

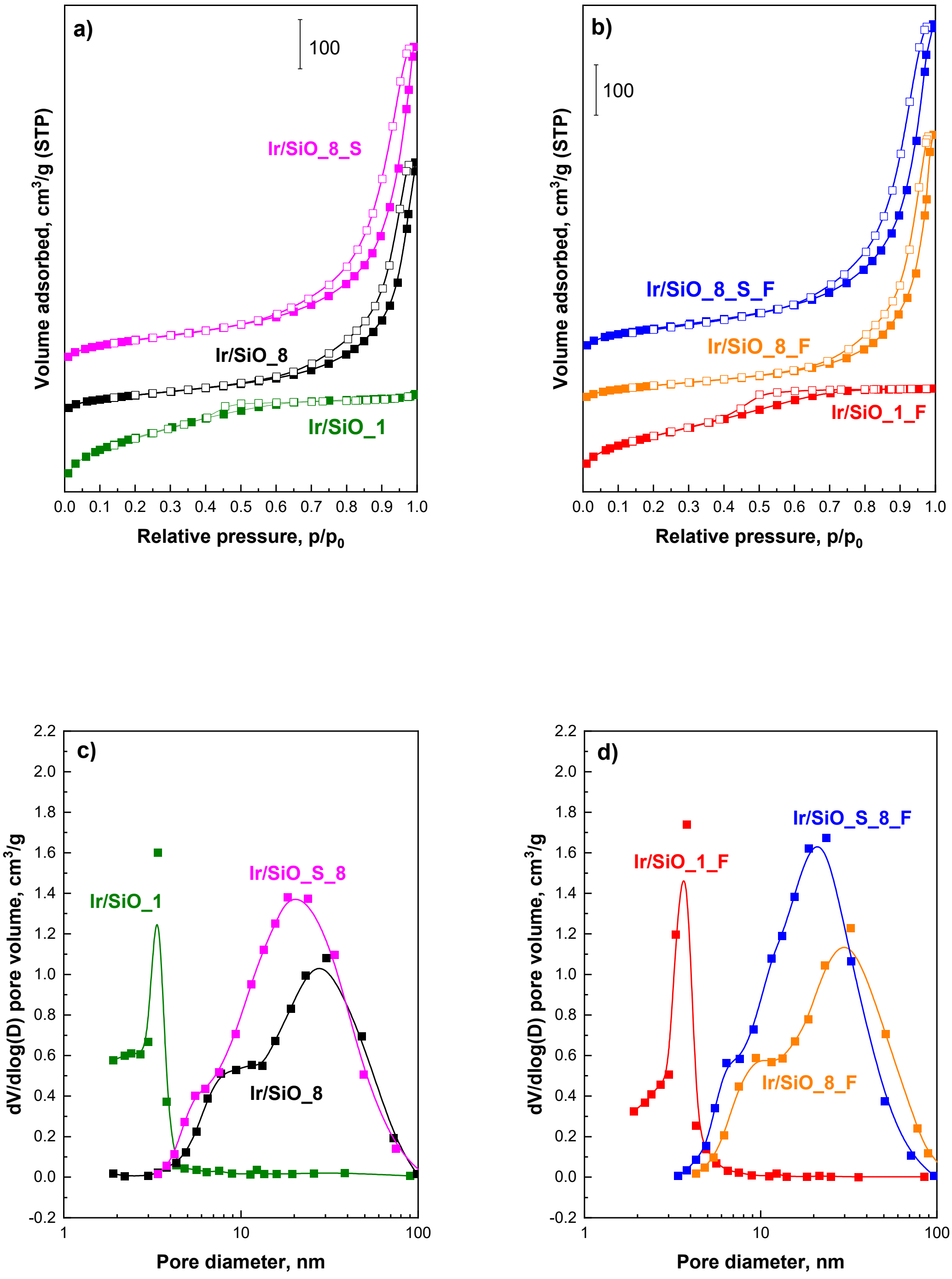

Figure S5. The effect of iridium deposition on the silica surface on the nitrogen adsorption-desorption isotherms **(a-b)** and the pore size distributions **(c-d)**.
